# Hepatitis C Virus Diversity and Evolution in the Full Open-Reading Frame during Antiviral Therapy

**DOI:** 10.1371/journal.pone.0002123

**Published:** 2008-05-07

**Authors:** Nathan A. Cannon, Maureen J. Donlin, Xiaofeng Fan, Rajeev Aurora, John E. Tavis, for the Virahep-C Study Group

**Affiliations:** 1 Department of Molecular Microbiology and Immunology, Saint Louis University School of Medicine, Saint Louis, Missouri, United States of America; 2 Department of Internal Medicine, Saint Louis University School of Medicine, Saint Louis, Missouri, United States of America; 3 Saint Louis University Liver Center, Saint Louis University School of Medicine, Saint Louis, Missouri, United States of America; National Institute for Communicable Diseases, South Africa

## Abstract

**Background:**

Pegylated interferon plus ribavirin therapy for hepatitis C virus (HCV) fails in approximately half of genotype 1 patients. Treatment failure occurs either by nonresponse (minimal declines in viral titer) or relapse (robust initial responses followed by rebounds of viral titers during or after therapy). HCV is highly variable genetically. To determine if viral genetic differences contribute to the difference between response and relapse, we examined the inter-patient genetic diversity and mutation pattern in the full open reading frame HCV genotype 1a consensus sequences.

**Methodology/Principal Findings:**

Pre- and post-therapy sequences were analyzed for 10 nonresponders and 10 relapsers from the Virahep-C clinical study. Pre-therapy interpatient diversity among the relapsers was higher than in the nonresponders in the viral NS2 and NS3 genes, and post-therapy diversity was higher in the relapsers for most of HCV's ten genes. Pre-therapy diversity among the relapsers was intermediate between that of the non-responders and responders to therapy. The average mutation rate was just 0.9% at the amino acid level and similar numbers of mutations occurred in the nonresponder and relapser sequences, but the mutations in NS2 of relapsers were less conservative than in nonresponders. Finally, the number and distribution of regions under positive selection was similar between the two groups, although the nonresponders had more foci of positive selection in E2.

**Conclusions/Significance:**

The HCV sequences were unexpectedly stable during failed antiviral therapy, both nonresponder and relapser sequences were under selective pressure during therapy, and variation in NS2 may have contributed to the difference in response between the nonresponder and relapser groups. These data support a role for viral genetic variability in determining the outcome of anti-HCV therapy, with those sequences that are more distant from an optimal sequence being less able to resist the pressures of interferon-based therapy.

**Trial registration:**

ClinicalTrials.gov NCT00038974

## Introduction

Hepatitis C Virus (HCV) infects over 170 million people worldwide and more than 4 million in the USA [1]–[3]. The costs related to HCV infection in the United States are over $700 million annually [4], and the impact of HCV infection is expected to rise over the next 20 years. Current therapy for HCV employs pegylated interferon α and ribavirin, but treatment clears the infection in only about half of patients infected with genotype 1, the most common genotype in the USA [5]–[7]. The reasons for failure of treatment are not fully understood, but host, virus, and immune response variables all correlate with response to therapy [5], [8].

HCV is a *Hepacivirus* in the *Flaviviridae* family. Its genome is a ∼9600 nucleotide, positive polarity single stranded RNA which contains a single large open-reading frame. The structural proteins include the core protein, which forms the viral capsid, and two surface glycoproteins, E1 and E2. Nonstructural proteins include a putative ion channel (p7), an autoprotease (NS2), a protease/helicase (NS3/4A), a putative organizer of the replication complex (NS4B), a pleiotropic regulatory protein (NS5A), and an RNA-dependent RNA polymerase (NS5B). An eleventh protein, the alternate reading frame protein, is encoded in the +1 frame of the core gene and is of unknown function [9]–[11].

HCV is highly genetically variable, with six different genotypes that have less than 72% homology at the nucleotide level. Each genotype is divided into multiple subtypes with 80-85% similarity. Isolates within each subtype are also extremely variable, with 8-12% divergence between isolates from independent patients [12]–[16]. Viral genetic variability contributes to differences in response to therapy because different genotypes respond to therapy at different rates; genotype 2 responds to six months of therapy over 80% of the time, while response in genotype 1 is about 50% after 12 months of treatment [6], [17]–[19]. Differences in viral genetic variability in discrete protein regions have also been linked to differences in response to therapy [15], [19]. High variation in the interferon-sensitivity determining region (ISDR) in NS5A has been associated with response to interferon-based therapy in some studies [20]–[23], and correlations between variations in the PKR-eIF2 phosphorylation homology domain (PePHD) in E2 and response to therapy have been noted [24]–[31]. Employing a full open-reading frame sequencing strategy, we have shown that high variation in genotype 1a NS3 and NS5A is associated with early response to therapy [32].

There are at least two different patterns by which HCV therapy can fail. Nonresponders have only minimal declines in viral titers, while relapsers have robust declines followed by a rebound in titers either during or after therapy. These different patterns could be affected by many factors including host genetics, immune response, and viral genetic differences [6], [8], [18], [19], [33]. Viral genetic differences could include either pre-therapy differences or differences that arise during treatment due to viral evolution in response to the pressures applied by therapy.

We previously reported that high pre-therapy inter-patient variability in NS3 and NS5A correlated strongly with early response to therapy in genotype 1a infected patients [32]. Here, we hypothesized that there would be differences in pre-therapy viral genetic variability between nonresponders and relapsers, and that HCV sequences in the relapsers would have higher rates of amino acid mutations than in nonresponders during therapy since nonresponder sequences were relatively resistant at the onset of therapy. To evaluate this hypothesis, we analyzed pre- and post-therapy full open-reading frame sequences from 20 participants in the Virahep-C clinical study of factors affecting response to antiviral therapy [8]; ten were nonresponders, and ten were relapsers. The inter-patient variability among the nonresponders and relapsers at pre-and post-therapy times was compared, and the evolution of the virus over the course of therapy was assessed.

## Results

### Experimental design and patient selection

**Figure 1:**
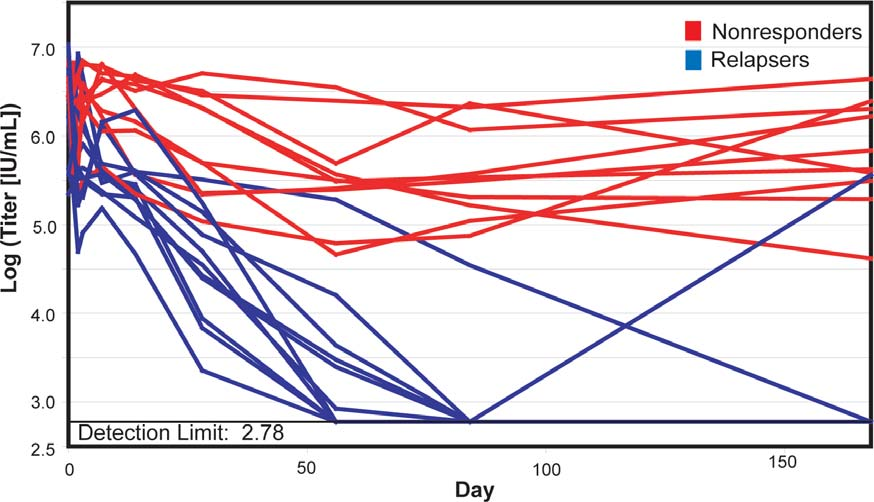
Viral titers in relapsers and nonresponders during the first 24 weeks of therapy. Viral titers are shown as the log(titers [IU/mL]) at each time point. All relapsers (blue) dropped to the detection limit. One of the relapsers had a rebound of viral titers during the first 24 weeks. All others had rebounds later in therapy. All nonresponders (red) declined by less than 2.1 log_10_.

The subjects in this study were chosen from the patients in the Virahep-C viral genetics study who failed pegylated interferon α and ribavirin therapy [32] infected with HCV genotype 1a for whom samples were available for sequencing 6 months post-therapy. Nonresponders had viral titers declines of ≤2.1 log_10_ IU/mL and absolute titers of ≥4.62 log_10_ IU/mL at nadir. Relapsers had declines in viral titers of ≥2.8 log_10_ and their absolute titers transiently dropped below the detection limit (2.78 log_10_ IU/mL) (Figure 1). The baseline characteristics for the two groups are shown in Table 1. There were no significant differences in baseline characteristics (p<0.05) in any characteristics except AFP, a nonspecific tumor marker, and the Ishak necroinflammatory score, an indicator of liver inflammation. Interestingly, pre-therapy HCV RNA levels were not predictive of the difference between relapse and nonresponse to therapy.

**Table 1:** Patient characteristics.

	Nonresponder^a^	Relapser^a^	p-value^b^
**Age (years)**	47.5 (45, 53)	48 (43, 49)	0.57
**Male**	8	8	1.00
**Body weight (kg)**	94.6 (89.4–103.4)	89.1 (77.6, 98.4)	0.22
**African-American**	5	4	1.00
**HCV RNA (log_10_ IU/mL)**	6.63 (6.45, 6.77)	6.52 (6.2, 6.68)	0.16
**ALT (U/L)**	71 (54, 104)	66.5 (54, 91)	0.96
**Albumin (g/dL)**	4.1 (4, 4.4)	4.2 (4.1, 4.6)	0.40
**Platelet count (1000)**	182.5 (161, 203)	204 (144, 225)	0.97
**AFP**	105 (95, 123)	65 (54, 83)	0.01
**Ishak necroinflamatory score (0–18)**	11.5 (11, 15)	7 (6, 11)	0.02
**Ishak fibrosis score**	3.5 (2, 4)	1.5 (1, 2)	0.09

a - numbers in brackets represent 25^th^ and 75^th^ percentile.

b - p-values are from permutation test based on 100,000 permutations, except for comparing proportion of males and proportion of African Americans for which Fisher's exact test was used.

### Conserved positional differences between nonresponders and relapsers

We first determined if there were consistent genetic differences at discrete amino acid positions between the nonresponders and relapsers. To do this, we created consensus sequences of the nonresponder and relapser sequences at 60% conservation levels for both pre- and post-therapy samples. The consensus sequences for the nonresponder and relapser alignments were then compared to determine if there were positions that differed consistently between the two phenotypes. For pre-therapy sequences, nine positions differed between nonresponders and relapsers (Table 2). In most of these cases, the dominant amino acid in one group was the second most abundant amino acid in the other group. In the post-therapy sequences, nine positions differed, five of which were also found in the pre-therapy data. All four positions that were novel in the post-therapy analysis resulted from a single amino acid change during therapy that caused the position to cross the 60% threshold. Likewise, the pre-therapy positions that were not consistently different in post-therapy samples were also all the result of single amino acid changes in the samples except position 394, which is within hypervariable region 1 (HVR1) in E2. The one exception to the presence of the dominant amino acid in one phenotype being present in the other group was at position 2283 [position 311 of NS5A, between the ISDR (237–276) and the SH3 domain (343–356)] in pre-therapy sequences [34]. Proline was found at this position in all but one of the nonresponders, which had an arginine. However, proline was also found in 40% of relapsers with the remaining 60% having glutamine.

**Table 2:** Amino acid differences between nonresponders' and relapsers' consensus sequences at ≥60% conservation.

Position (Polyprotein)	Protein	Position	Nonresponder		Relapser	
			Primary	Secondary	Primary	Secondary
**Pre-therapy**						
394	E2	11	H	R(2),K(1),Y(1)	R	H(2),F(1),S(1)
787	p7	41	V	A(4)	A	V(3),T(1)
879	NS2	70	V	A(2)	A	V(3)
1444	NS3	418	F	Y(2)	Y	F(3)
2283	NS5A	311	P	R(1)	Q	P(4)
2373	NS5A	401	P	S(4)	S	P(2)
2413	NS5A	441	D	G(4)	G	S(2),D(1)
2518	NS5B	98	K	R(4)	R	K(4)
2720	NS5B	300	Q	R(4)	R	Q(3),L(1)
**Post-therapy**						
879	NS2	70	V	A(2)	A	V(4)
929	NS2	120	A	V(3),I(1)	V	A(4)
1412	NS3	386	I	V(2),L(2)	V	I(4)
1444	NS3	418	F	Y(3)	Y	F(4)
1686	NS4A	29	I	V(4)	V	I(3)
2373	NS5A	401	P	S(3)	S	P(2),T(1)
2411	NS5A	439	G	E(4)	E	G(1)
2518	NS5B	98	K	R(4)	R	K(4)
2720	NS5B	300	Q	R(4)	R	Q(3),L(1)

Numbers in parentheses indicate the frequency of the secondary amino acids in the ten sequences.

Therefore, there were only a few amino acids that differed consistently between the nonresponder and relapser sequences, and these differences were relatively conserved after therapy. However, the weak degree of conservation at these positions, the common presence of the alternate amino acid from the opposing phenotype, and the conservative nature of the alternate amino acids at these positions all argue against variation at these positions playing a major role in determining if the patient was a nonresponder or relapser during therapy.

### Inter-patient genetic diversity among the nonresponder and relapser sequences

Since there were no amino acid positions at which genetic differences strongly correlated with nonresponse or relapse, we next examined the groups of sequences to determine if there were differences in inter-patient diversity between the nonresponders and relapsers that correlated with the response pattern. Diversity differences were measured by comparing the numbers of amino acid variations relative to a reference sequence and by measuring differences in the average protein distances within the two groups.

**Figure 2:**
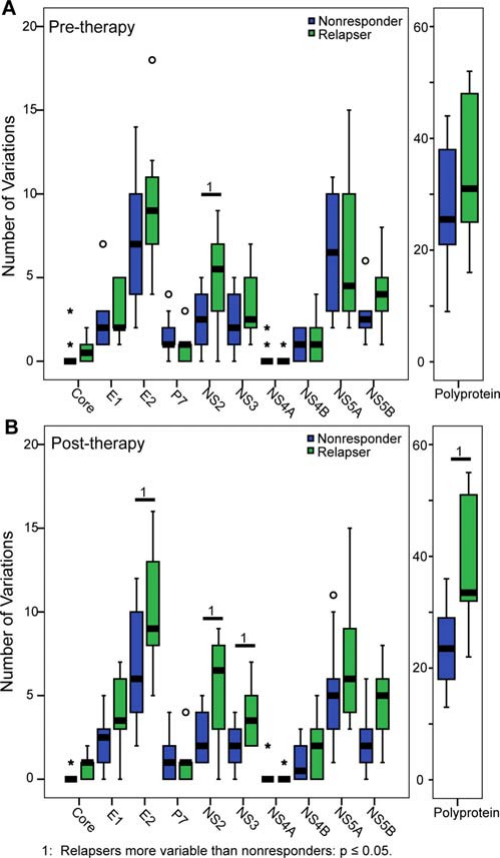
Unique amino acid variability among nonresponders and relapsers. Amino acid variations found exclusively in one response class but not in the other were compared between nonresponders and relapsers. Statistical significance of the difference in the number of variations was compared using the Mann-Whitney test. A) Pre-therapy. B) Post-therapy. The line represents the median value, and the box represents the 25–75% range. Whiskers represent samples within 1.5 box lengths, and the ° and * represent outliers between 1.5 and 3 box lengths and beyond 3 box lengths respectively.

First, each sample was aligned against an external genotype 1a reference sequence [32], and positions of variation relative to the reference were identified for each sample. Variations were classified as unique to either relapsers or nonresponders if the variation was observed in one group of sequences but not in the other. In pre-therapy sequences, the polyproteins had similar numbers of unique variations in nonresponders and relapsers (Figure 2A). To determine if the variations were evenly distributed throughout the polyprotein, we compared the number of variations in each gene. Relapsers had significantly more unique variations in NS2 than the nonresponders by the Mann-Whitney test (p = 0.048). All other proteins had similar numbers of unique variations between the nonresponders and relapsers (p≥0.05). When the numbers of unique variations in the post-therapy polyproteins were compared, relapsers had more unique variations than nonresponders (p = 0.006) (Figure 2B). Examination of the individual genes revealed that there were more unique variations in the relapsers in E2 (p = 0.033), NS2 (p = 0.027), and NS3 (p = 0.045).

**Figure 3:**
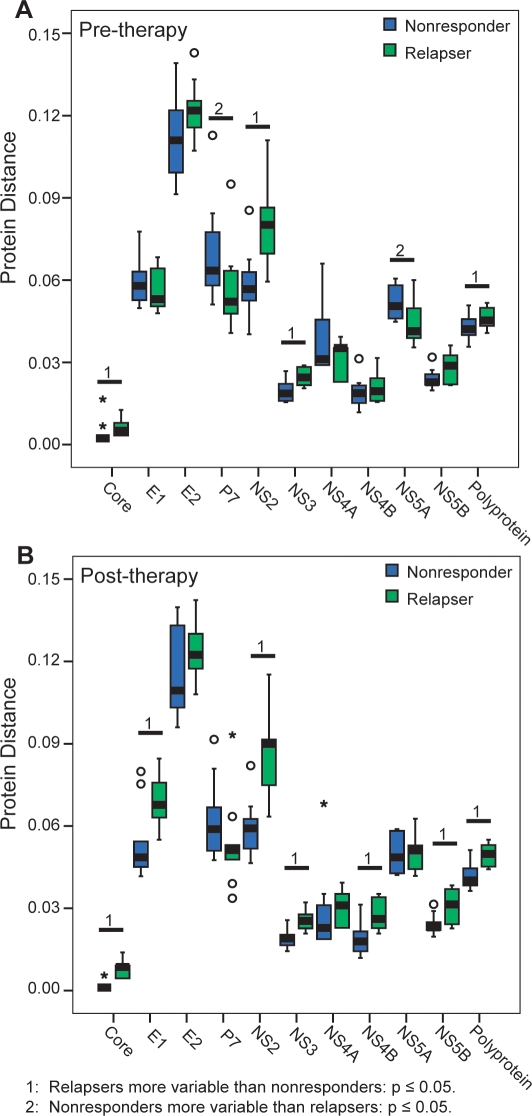
Protein distance among the nonresponder and relapsers sequences. The mean protein distance among the nonresponders and relapsers was compared, and the statistical significance was evaluated using the Mann-Whitney test. A) Pre-therapy. B) Post-therapy.

As a second measure of sample diversity, we compared the average pair-wise protein distance within each group. The pair-wise protein distances among the nonresponders and relapsers were determined separately, and the mean protein distance for each sample relative to the other nine nonresponder or relapser sequences was determined. We then compared the average distances among the nonresponders and relapsers and determined their statistical significance using the Mann-Whitney test. In the pre-therapy data (Figure 3A), relapsers had higher intra-group genetic distances than nonresponders for the polyprotein (p = 0.049). When individual proteins were compared, the relapsers had a higher average distance in core (p = 0.007), NS2 (p = 0.003), and NS3 (p = 0.009), while the nonresponders had higher average distance in P7 (p = 0.041) and NS5A (p = 0.019). When the post-therapy samples were compared (Figure 3B), relapsers had higher distances in the polyprotein (p = 0.003), and in core (p = 0.001), E1 (p = 0.010), NS2 (p = 0.001), NS3 (p = 0.001), NS4B (p = 0.004), and NS5B (p = 0.007). Therefore, the relapsers had a higher overall protein distance than the nonresponders in both pre- and post-therapy sequences, the differences in protein distance were broadly distributed through the polyprotein, and the differences were more pronounced in post-therapy samples.

**Figure 4:**
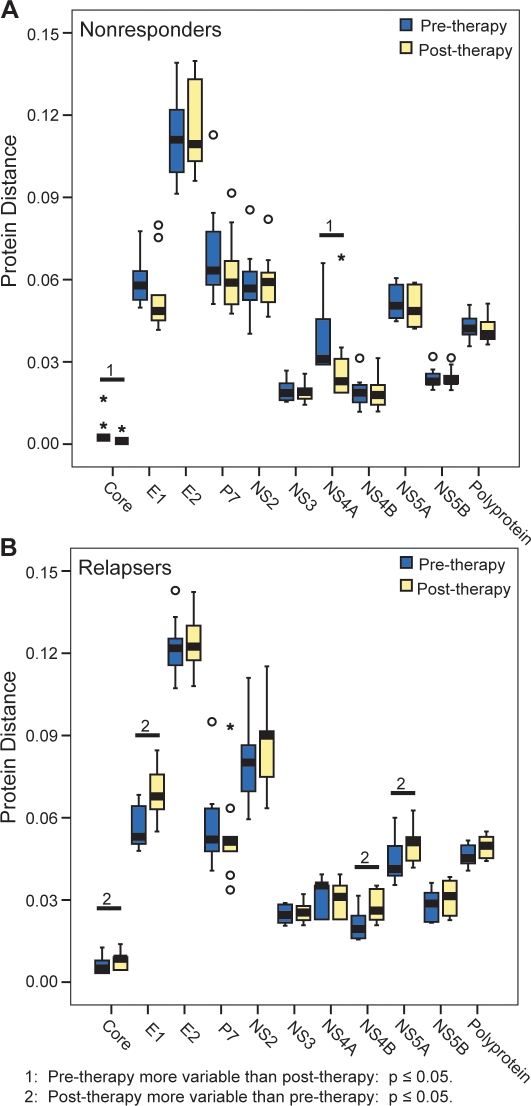
Protein distance in pre- and post-therapy samples. Comparison of pre- and post-therapy protein distances in A) nonresponders and B) relapsers. Statistical significance was determined using the Mann-Whitney test.

We next asked whether the greater differences between nonresponders and relapsers in the post-therapy analysis were due to changes in sequences from the nonresponders, relapsers, or both by comparing the pre- versus post-therapy protein distances among the nonresponders and relapsers (Figure 4). The protein distance among nonresponders declined significantly in core (p = 0.006) and NS4A (p = 0.027) in the nonresponders during therapy, while the other proteins did not change significantly. Relapsers had a different pattern. Core (p = 0.046), E1 (p = 0.006), NS4B (p = 0.023), and NS5A (p = 0.041) had statistically significant increases in protein distance during therapy, and many of the other proteins, including the polyprotein, had increases that did not reach statistical significance. Overall, protein distances declined slightly in nonresponders during therapy, whereas they increased substantially in relapsers.

**Figure 5:**
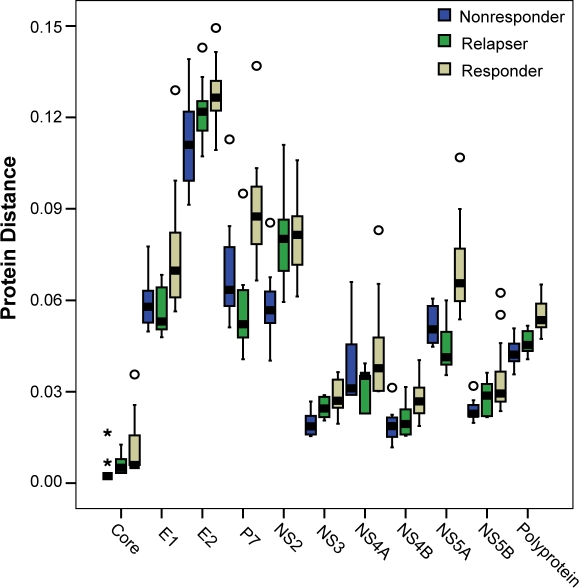
Protein distance in nonresponders, relapsers, and responder pre-therapy sequences. Comparison of the protein distances in three-phenotypes of patients.

Finally, the inter-patient protein distance of pre-therapy sequences among nonresponders, relapsers, and responders was compared to the distance among sustained viral responders to antiviral therapy in the Virahep-C study [32] to determine how the patterns observed in nonresponders and relapsers compared to sequences that were responsive to pegylated interferon α and ribavirin therapy. The intra-group distances of responders were plotted in Figure 5 along with the nonresponders and relapsers. Two patterns were most common. First, in the polyprotein, core, E2, NS3, NS4B, and NS5B, the responders had the highest distances between samples, relapsers were intermediate, and nonresponders had the lowest distances. The second pattern was observed in E1, P7, NS4A, and NS5A, where the relapsers were similar to the nonresponders but the responders had higher distances. The single exception to these two patterns was in NS2, where the relapsers had distances similar to the responders but the nonresponders were significantly lower. When analyzed using ANOVA, there were differences in all proteins (p<0.005). These data indicate that a spectrum of variability exists among the response classes, with responders being the most variable, relapsers being intermediate, and nonresponders being the least variable. The most notable exception to this general pattern was NS2, where relapsers resembled responders.

### Evolution of viral sequences during therapy

**Figure 6:**
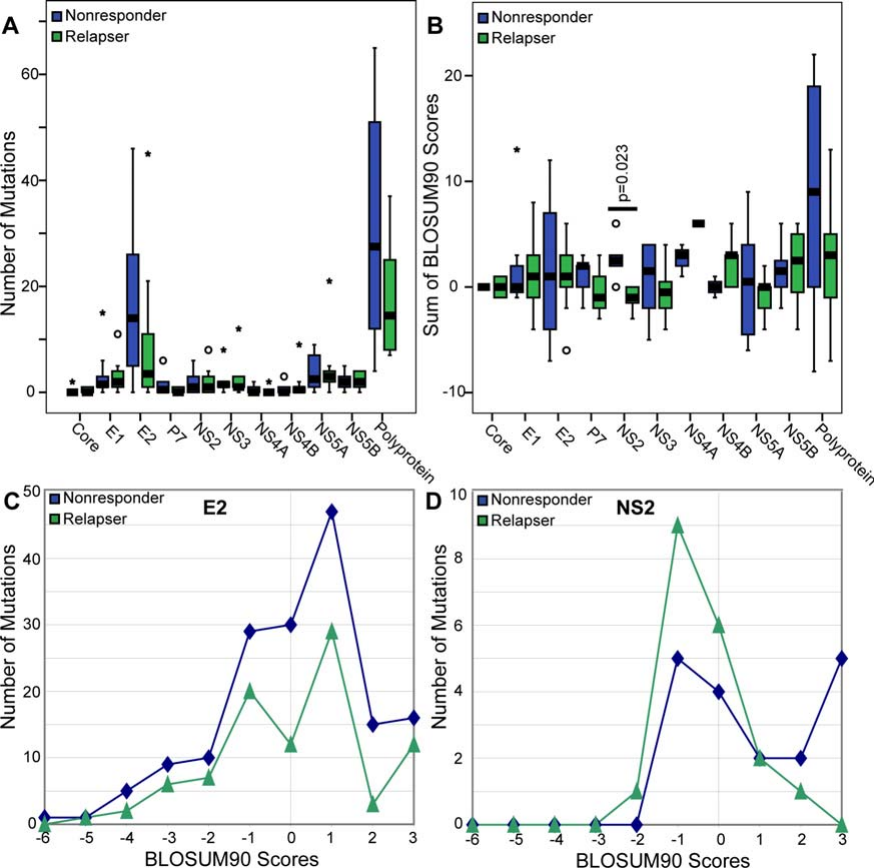
Mutations in nonresponder and responder sequences over the course of therapy. A) The number of changes in nonresponders and relapsers were compared using Mann-Whitney test. One relapser does not appear on the chart (113 changes). B) The sums of the BLOSUM90 score for all of the changes occurring in a given sample were compared between nonresponders and relapsers. Totals were compared using a Kolmogorov-Smirnov test. C) Distribution of BLOSUM90 scores for E2. D) Distribution for BLOSUM90 scores for NS2.

If differences in viral genetic variability contribute to the ability of the virus to withstand the pressures induced by therapy, those isolates that survive therapy would be relatively resistant to the effects of the drugs whereas those that do not survive would be sensitive. Resistance could either be present initially or evolve during therapy. Therefore, we hypothesized that there would be a difference in evolution between the two groups of sequences, with relapsers evolving to become more resistant to therapy while nonresponders would evolve less because they were initially relatively resistant. To test this hypothesis, paired pre- and post-therapy sequences from each patient were aligned, and mutations at the amino acid level that occurred during therapy were identified. Contrary to our expectations, nonresponders had more mutations in the polyprotein than the relapsers (Figure 6A), but this difference was located entirely in E2. Two regions of E2 were analyzed in detail due to their potential to affect the outcome of therapy: HVR1 and PePHD. HVR1 (amino acids 384–410) encodes a decoy B-cell epitope [35]. In HVR1, the nonresponders had 56 mutations in nine of ten samples, while relapsers had 32 mutations in six of ten samples (p>0.1 by the Mann-Whitney test). The PePHD region (amino acids 659–670) can bind to PKR and inhibit its activity [36], [37], but there were no changes in the PePHD region in either the nonresponders or the relapsers.

To assess the likelihood that these mutations may have altered the activity of the proteins, the BLOSUM90 scores for each mutation were evaluated. BLOSUM scores are log-odds ratios of amino acid substitutions. Substitutions which occur more often than expected have positive scores and reflect conservative changes, and substitutions which occur less often than expected have negative scores and reflect non-conservative changes. For all proteins except NS2, the scores in nonresponders and relapsers were not significantly different. In NS2, relapsers had significantly lower scores than nonresponders by the Kolmogorov-Smirnov test (p = 0.023) (Figure 6B). We next examined the distribution of the BLOSUM90 scores for each gene. Most of the plots for relapsers and nonresponders were quite similar. An example is for E2 in Figure 6C, where there was a difference in the total number of mutations in relapsers and nonresponders, but the BLOSUM90 score distribution was similar. However, differences in the score distribution were evident for NS2 (Figure 6D). All of the mutations in nonresponders had scores ranging from −1 to 3, with a relatively even distribution throughout that range. In contrast, most of the scores for relapsers ranged from −2 to 0. Together, these data indicate that the mutations in the relapsers were more likely to affect the function of NS2 than the mutations in the nonresponders.

### Locations of mutations on the known protein structures

Crystal structures have been determined for all or part of NS2 [38], NS3 [39], NS5A [40], and NS5B [41]. Mutations that occurred during therapy were mapped onto these structures, and the distribution of changes was analyzed by visual inspection and comparisons to known functional sites and secondary structures on the protein. No clear differences were noted in the distributions of mutations from nonresponders and relapsers.

### Patterns of positive selection during therapy

**Figure 7:**
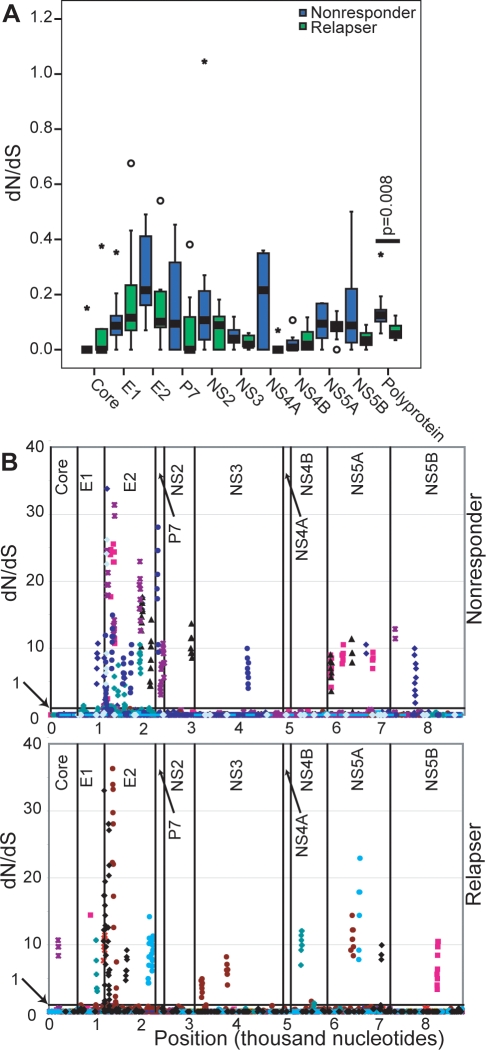
The dN/dS is similar in nonresponders and relapsers. A. Comparison of dN/dS ratios between nonresponders and relapsers. dN/dS was determined by the Nei-Gojobori method. B. dN/dS in ten codon windows as determined by SWAPSC using the Li-Kimura method. Each sample is represented by a different color and each dot indicates a specific 10 codon window. Regions of positive selection are overlapping 10 codon windows with dN/dS >1, and are denoted as a collection of points on the graph forming an upward spike.

To determine if there was a difference in the degree or pattern of positive selection between nonresponders and relapsers during therapy, we measured the nonsynonymous to synonymous substitution ratio (dN/dS) by the Nei-Gojobori method in each patient and compared the ratios between nonresponders and relapsers using the Mann-Whitney test. For the entire polyprotein, nonresponders had higher dN/dS ratios than relapsers (p = 0.008) (Figure 7A). However, this difference was distributed widely throughout the viral genome, and hence no individual gene achieved statistical significance.

Many different selective pressures could be applied to the HCV genome by the pleiotropic effects of interferon and ribavirin. Negative selection maintains critical functions in many positions, and positive selection would affect specific regions of proteins associated with differences in response to the therapy. However, because all genes in the HCV genome are linked, positive selection at any given site could co-select neutral variations throughout the genome. Therefore, to determine if there were small regions of the protein that were under positive selective pressure, we examined the dN/dS ratio by the Li-Kimura method in overlapping windows of 10 codons. Seven of ten nonresponders and seven of ten relapsers had regions of positive selection (dN/dS >1), and these regions were distributed throughout the viral genome (Figure 7B). The polyproteins of nonresponders had 43 regions of positive selection while relapsers had 23 regions, but this difference was not statistically significant. Furthermore, there were no apparent differences between the two groups in any gene except E2, where nonresponders had more regions of positive selection in E2 than the relapsers (27 vs. 10) (p>0.05). Therefore, most nonresponder and relapser sequences evolved in response to the selective pressures induced by therapy, but E2 changed more in nonresponders than in relapsers.

### Quasispecies breadth

**Figure 8:**
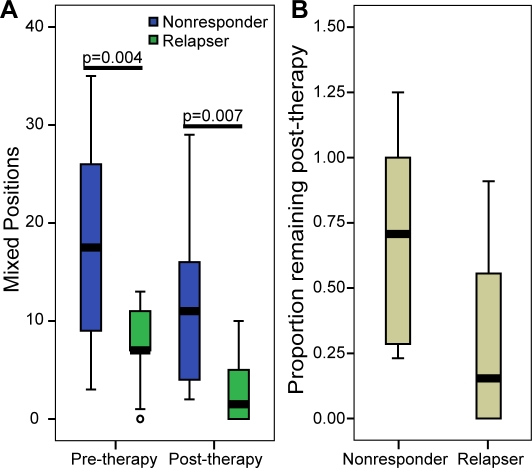
Quasispecies breadth is lower in relapser than nonresponder sequences. Nonresponder and relapser sequences were analyzed using Phred and positions at which the dominant nucleotide was present at <80% frequency were determined. A) Number of positions of quasispecies diversity in nonresponders and relapsers in pre- and post-therapy samples. B) Proportion of positions of mixed nucleotides in post-therapy samples relative to pre-therapy samples. One nonresponder (3.22) and one relapser (10.00) are not shown. The relapser is the same as was removed from Figure 3A.

HCV replicates as a quasispecies, but our analyses were performed on the consensus sequence. Therefore, to determine if there were differences in the quasispecies breadth between the nonresponder and relapser sequences, we evaluated the prevalence of mixed base positions in the sequence traces of the uncloned sequencing templates using the method developed by A. F. Poon [42]. This method cannot identify a dominant quasispecies sequence, but it can measure the breadth of the quasispecies at each nucleotide position by identifying the positions where the dominant nucleotide is present at less than 80% frequency. Patterns of quasispecies variation in E2 have been well characterized in many studies, especially in HVR1 [43]–[47]. Thus, we compiled the sequences from E2 for each sample, and compared the numbers of mixed bases found in nonresponders versus relapsers using the Mann-Whitney test. The nonresponders had more mixed base positions than relapsers both pre- and post-therapy (p = 0.004 and p = 0.007 respectively) (Figure 8A).

Genetic bottlenecks decrease the genetic breadth within a population, and a decrease in the diversity of both nonresponders and relapsers was observed, consistent with both groups passing through a bottleneck. However, the decline in diversity would be expected to be greater in those populations that pass through a tighter bottleneck. Thus, we expected the proportions of mixed bases retained in the post-therapy samples relative to pre-therapy samples to be lower in relapsers than in nonresponders. When the proportions of mixed bases were compared, the relapsers had the expected greater decline in mixed bases from pre- to post-therapy samples than the nonresponders, but this difference was not statistically significant by the Mann-Whitney test (p = 0.079) (Figure 8B).

## Discussion

Failure of interferon plus ribavirin therapy for HCV can occur in two different patterns: nonresponse and relapse. HCV is highly diverse genetically, and this diversity could affect how the virus responds to therapy. Furthermore, HCV can evolve rapidly, and hence therapy could drive evolution of the virus by selecting relatively resistant variants. We previously found that high genetic diversity in genotype 1a pre-therapy NS3 and NS5A sequences correlated robustly with response to therapy [32]. The previous study addressed variability related to both response to therapy and the race of the patient. Here, we employed the pre- and post-therapy sequences from genotype 1a Virahep-C treatment failures to evaluate both the extent to which variations in viral protein sequences may affect the pattern of failed therapy and to determine the effects of failed therapy on the viral sequences. Issues of variability associated with the race of the patient were not addressed in this study because the low number of samples available does not provide enough power to make useful comparisons.

To evaluate how the viral protein sequences changed during treatment, we examined the number and pattern of amino acid mutations that occurred in each sequence during therapy. We expected to find a relatively high mutation frequency due to HCV's high genetic plasticity, but contrary to our expectations, the 0.9% mutation frequency observed in the samples was relatively small in relation to the variability among independent HCV isolates (10–12%), and was in the range of quasispecies variability typically found in a given individual (∼1–4%). Furthermore, about one third of the mutations we observed were in E2, especially in HVR1, so the mutation rate of the HCV ORF outside E2 was just 0.59%. Thus, a primary conclusion of this study is that the HCV consensus sequence is relatively stable during failed antiviral therapy. Most previous studies of HCV evolution during therapy have focused on small regions of the genome, primarily in NS5A and E2, and especially on the highly variable regions in these genes [23], [48]–[52]. These regions were chosen because their high variation makes them ideal for evolutionary analyses. However, focusing on theses regions had the unintended effect of helping to form the perception that HCV sequences are similarly mutable throughout the genome.

The second major conclusion from this study was that genetic differences in NS2 correlated with the pattern of failed response to interferon-based therapy. These correlations were evident in the number of pre-therapy unique variations (Figure 2), the pre-therapy protein distance (Figure 3), and the nature of mutations that occurred during therapy (Figure 6B and D). Relapsers had higher variability in NS2 than nonresponders in pre-therapy samples, and mutations that occurred in NS2 over the course of therapy were more likely to affect the function of the protein in relapsers than in nonresponders. Therefore, variability in NS2 may help determine whether a patient will be a nonresponder or relapser. However, this possibility is difficult to interpret at a functional level because the roles of NS2 in viral replication and pathology are poorly understood. NS2 has been shown to inhibit the interferon response when expressed in cells [53], and the observed differences in variability could lead to differences in the effectiveness of this inhibition. Alternatively, since NS2 is involved in protein processing and is required for virion formation [54], variability could also affect the effectiveness of viral protein processing and virus production. Further study of NS2, including identifying possible cellular targets, may clarify how NS2 affects response to interferon-based therapy.

Despite the overall stability of the HCV sequences, a substantial number of mutations did occur during failed therapy, but no significant difference was observed in the number of mutations between the nonresponders and relapsers (Figure 6A). We had hypothesized that relapsers would evolve more than nonresponders as the virus adapted to the pressures of therapy. This was not observed at the level of the number of mutations, possibly because both groups passed through at least a weak bottleneck. However, despite equivalent numbers of mutations in the relapser and nonresponder groups, the overall intra-group genetic distance in the relapsers increased while it did not change in nonresponders (Figure 4). Therefore, the mutations in relapsers created new sequences, whereas those in nonresponders largely alternated between sequences already present within the group.

To determine which sequence motifs may have been under positive selection during therapy, we examined the dN/dS ratio in a sliding window of ten codons. Many regions of strong positive selection were observed (Figure 7B), but the number and distribution of regions of positive selection were similar in the nonresponder and relapser sequences. This implies that both nonresponders and relapsers evolved to similar degrees under the pressures induced by therapy and that the targets of the selective pressure were broadly distributed throughout the polyprotein. The exception to this pattern was in E2, where there were more regions of positive selection in nonresponders than in relapsers. As E2 is a primary target of humoral immune responses, this difference may be due to the difference in neutralizing antibody titers throughout therapy. Brown et al. showed that E2 and not E1 evolve in chronically infected patients in solvent exposed regions [55], and our data show that there were differences in evolution of the nonresponder and relapser sequences. Since the nonresponders had relatively high viral titers throughout therapy, the humoral immune response may have been be constantly stimulated by a relatively high antigen load, leading to an evolving humoral pressure. In relapsers, viral titers declined below the detection limit, and the humoral immune response may have declined during therapy due to the drop in antigen load. These analyses compared pre- and post-therapy sequences, and the patterns of evolution observed in samples during therapy may be different than those that were prevalent in post-therapy samples. Therefore, studies of samples from sustained viral responders, nonresponders, and relapsers at early time-points during therapy, such as 2 or 4 weeks, could be useful in further understanding the evolution of these groups in response to interferon-based therapy. However, the relatively small number of changes observed in the viral consensus sequence between the pre- and post-therapy time points implies that a detailed quasispecies analysis over the early phases of therapy would be needed to substantially advance this understanding.

Genetic bottlenecks can cause a constriction of the genetic variability within a population. In HCV, this is reflected in the breadth of the quasispecies within an individual. We expected the difference in the strength of the bottlenecks experienced by nonresponder and relapser groups to cause a greater decline in the quasispecies breadth in the relapsers. We found that the intra-patient quasispecies breadth declined in both nonresponders and in relapsers, and that the decline in relapsers was greater than in nonresponders, but this difference did not reach statistical significance (Figure 8B). Other groups have shown that the breadth of the quasispecies is correlated with response to interferon-based therapy [44], [46]. Our study indicates that changes in the breadth of the quasispecies also correlated with the difference in response between nonresponders and relapsers.

This study was designed to assess that role of HCV genetic variation at the protein level on outcome of therapy. Variability in the RNA itself can also predicted to affect the response to therapy by altering the RNA structure or the interactions of the RNA with host proteins and/or the viral replication machinery. RNA elements associated with protein binding could occur anywhere in the viral RNA, but they are most likely to occur in the 3′ and 5′ UTRs since these areas are known to contain the promoters for viral replication and the viral internal ribosome entry site. The sequences obtained for this study include part of the UTRs, but these sequences are of varying length, and in some cases are absent. This precludes meaningful analysis of these samples outside the ORF.

This study is the largest examination to date of genetic changes in the full-length viral open-reading frame during interferon-based therapy, and it is the first study comparing nonresponders to relapsers in genotype 1a. Previous studies have examined the changes in patient samples over the course of therapy. Enomoto identified the ISDR [56] by examining pre- and post-therapy sequences in full-length sequences from three nonresponding genotype 1b infected patients. The three patients also had many mutations scattered elsewhere throughout the structural and nonstructural genes. We saw similar patterns of mutations in the 1a sequences. Other studies of evolution during therapy focused on smaller portions of the genome. Vuillermoz examined the changes in genotype 1b responders, nonresponders, and breakthrough patients in E2, NS5A, and NS5B [57]. They showed higher mutation rates in responders in the V3 region of NS5A (amino acids 2356-2379) and conservation of the PePHD region in E2 in all samples. We also found no mutations in the PePDH region, but unlike Vuillermoz, we did not find a difference between nonresponders and relapsers in the V3 region. Differences in the observed numbers of mutations between our study and the Vuillermoz study could be due to the different genotypes studied or the different definitions of the response types analyzed. Evolution of HCV during therapy has also been noted in NS5B [58], NS5A [23], [50], [52], and in the structural proteins, especially HVR1 [48]–[52]. While correlations between diversity and evolution between relapsers and nonresponders were noted in some of these studies, others showed no difference between the groups. We did not observe significant differences in NS5A or NS5B between the nonresponders and relapsers but we did find many regions of positive selection in E2 as well as HVR1, similar to earlier studies.

Genetic variability between HCV genotypes can lead to difference in response to therapy (e.g. genotype 1 vs. 2), and we have shown that variability differences are also associated with early response to therapy within a given viral subgenotype [32]. Here, we divided the nonresponders into two phenotypes, nonresponders and relapsers, and found a spectrum of diversity associated with failed response to antiviral therapy, where relapsers fell between responders and nonresponders. We interpret this pattern to indicate that viral variability forms a continuum from sequences that are close to an “optimal” sequence to sequences that are more divergent. The optimal sequence would be most resistant to the effects of therapy, and the degree of resistance would decline with genetic distance from the optimum. Those samples that were furthest from the optimum sequence would be unable to withstand the super-physiological interferon response induced by therapy, and hence would be cleared. Therefore, although variability of the virus is clearly not the only factor affecting response to therapy, it appears to play an important role in determining the pattern of response of HCV to interferon-based therapy. Further sequencing of isolates that are nonresponders to therapy and characterization of these sequences in in vitro studies could reveal this optimum sequence, and in vitro studies of this sequence could reveal how HCV inhibits the type 1 interferon response. Understanding how the HCV proteins are involved in resistance to interferon and ribavirin could identify new drug targets that improve or replace the current therapy.

## Materials and Methods

### Virahep-C

Virahep-C was a multi-center clinical study of peginterferon α-2a and ribavirin therapy in treatment naïve patients chronically infected with HCV genotype 1 [8]. Virahep-C included 205 Caucasian American and 196 African American participants, all of whom were treated with peginterferon α-2a (Pegasys™, Roche Pharmaceuticals; 180 µg weekly by self-administered subcutaneous injection) and ribavirin (Copegus™, Roche Pharmaceuticals; 1000 mg/day for those <75 kg and 1200 mg/day for those ≥75 kg, orally). Treatment was for 24–48 weeks depending on detection of viral titers at 24 weeks. Serum RNA levels were quantified as described previously [8], and the primary outcome was sustained viral response, undetectable viremia at 24 weeks post-therapy. All patients gave written informed consent to the Virahep-C study and its integral basic science studies, and this project was approved by the Saint Louis University Institutional Review Board. The CONSORT checklist and CONSORT flow chart are available as supporting information; see Figure S1 and Checklist S1.

### Sequencing

Consensus sequences for the full HCV ORF were obtained by direct sequencing of overlapping nested RT-PCR reactions as described previously [59]. Pre-therapy sequences were determined from samples taken just prior to the beginning of therapy. Post-therapy sequences were determined from samples collected 6 months following cessation of therapy. Post-therapy sequences were generated using conditions identical to those used in the pre-therapy samples to minimize amplification bias. When post-therapy sequences could not be obtained using the conditions previously employed for the pre-therapy samples [32], pre-therapy samples were resequenced using the primers and conditions that were used to amplify the post-therapy samples. When necessary, an alternate amplification method developed by Fan et al. involving long RT-PCR was used to amplify both pre- and post-therapy samples [60]. Pre-therapy samples were resequenced for samples 1013, 1030, 2011, 2027, 4025, 4035, 5009, 6018, 7002, 7003, 7040, 7041, and 7043. These sequences differed by <0.1% at the amino acid level from our previously reported sequences [32]. This amplification bias is within the 0.6% we reported for HCV genetic analyses and represents alternate samplings of the quasispecies spectrum [59]. The analysis included all but the final 56 amino acids of the open-reading frame because sequencing of the full ORF was not possible for 11 samples. The sequences have been deposited in Genbank, and are listed in Table 3.

**Table 3:** Patient numbers, response class, and GenBank accession numbers for pre- and post-therapy sequences.

Patient	Response*	Pre-therapy	Post-therapy
1013	N	EU362899	EU362876
1024	N	EF407428	EU362877
1030	N	EU362900	EU362878
2005	R	EF407452	EU362879
2011	R	EU362901	EU362880
2027	R	EU362902	EU362881
4025	R	EU362903	EU362883
4035	N	EF407453	EU362884
5009	N	EU362904	EU362885
5014	N	EF407435	EU362886
6020	R	EF407450	EU362887
6025	N	EU362905	EU362888
6030	R	EU362906	EU362889
7002	R	EU362907	EU362890
7003	N	EU362908	EU362891
7012	N	EF407438	EU362892
7040	N	EU362909	EU362893
7041	R	EU362910	EU362894
7043	R	EU362911	EU362895
7046	R	EF407433	EU362896

* N, nonresponders. R, relapsers.

### Sequence analysis

Amino acid sequences were deduced from nucleic acid sequences. Sequence alignments were done with ClustalW. The ARF gene was not analyzed due to differences in length of the protein in individual isolates. Amino acid positions that varied relative to the genotype 1a population consensus sequence [32] were identified using Mutation Master [61]. The genotype 1a consensus was derived from all 12 full-length ORFs in the Los Alamos National Laboratory and European HCV database [62] in April, 2005 that were from different patients, plus 5 additional 1a ORFs we sequenced from non-Virahep-C cohorts. The mean genetic distance was calculated using the p-distance algorithm in the MEGA3 DNA analysis package [63]. dN/dS ratios at the gene and whole-ORF levels were calculated with the MEGA3 DNA analysis package using the Nei-Gojobori method. dN/dS ratios in small windows were determined using SWAPSC using the Li-Kimura method [64].

### Quasispecies breadth

The sequence, quality, and polymorphisms in trace files of the first 1064 nucleotides of E2 were compiled for all 20 samples using Phred [65], [66]. Each trace file was converted into a single sequence file using a script provided by Dr. Art Poon [42] with positions where the dominant nucleotide was present at less than 80% maximum being indicated as polymorphisms. The numbers of mixed bases were determined using Clone Manager (Sci-Ed Software).

### Statistical analyses

The average genetic distance and numbers of unique variations between samples were compared using the Mann-Whitney test. BLOSUM90 scores were compared using the Kolmogorov-Smirnov test. A p-value of ≤0.05 was considered statistically significant. Statistical analyses were performed using SAS software or SPSS v. 13.0.

## Supporting Information

Checklist S1 CONSORT type checklist for the viral evolution study within the Virahep-C study.
(0.05 MB DOC)

Figure S1 CONSORT flowchart. A depiction of how patients were selected from the main Virahep-C study for this study on viral evolution.
(5.63 MB DOC)
