# Supplementary figures and images for: Hepatitis C Virus Diversity and Evolution in the Full Open-Reading Frame during Antiviral Therapy
Source: PLoS One. 2008 May 7;3(5):e2123. doi: 10.1371/journal.pone.0002123 (PMC2373758; doi:10.1371/journal.pone.0002123)

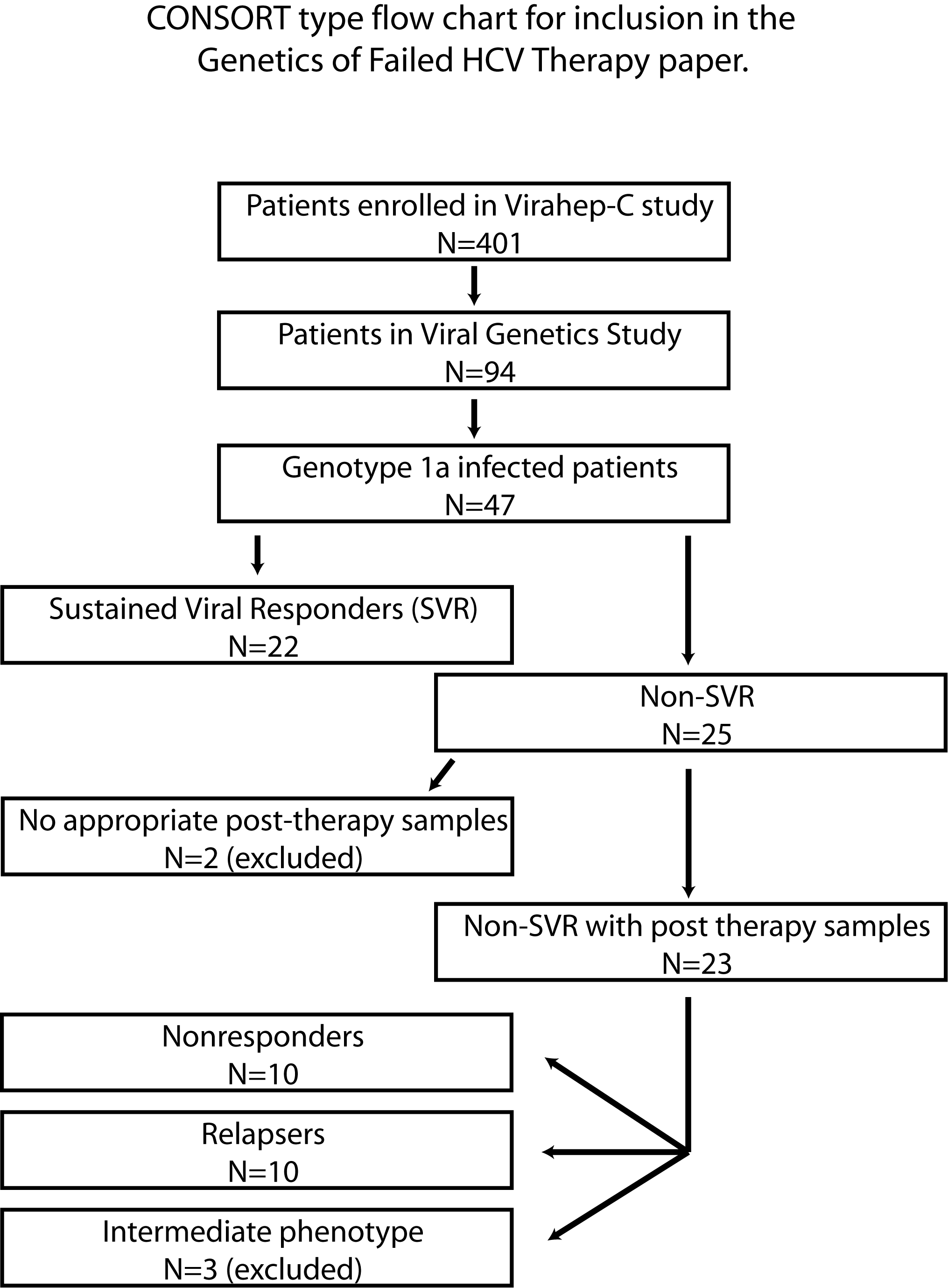

Supplement: Figure S1 — CONSORT flowchart. A depiction of how patients were selected from the main Virahep-C study for this study on viral evolution. (5.63 MB DOC) [file pone.0002123.s002.tif]
